# Development of a cell-type cylindrical carrot seeder

**DOI:** 10.1016/j.heliyon.2024.e39605

**Published:** 2024-11-06

**Authors:** Marvin T. Valentin, Keithler M. Pagnas, Roger Lee M. Suclad, Algirdas Jasinskas, Rolandas Domeika, Egidijus Šarauskis

**Affiliations:** a Department of Agricultural and Biosystems Engineering, College of Engineering, Benguet State University, Km. 5, La Trinidad, 2601, Benguet, Philippines; b Department of Applied Bioeconomy, Wrocław University of Environmental and Life Sciences, 51-630, Wroclaw, Poland; c Engineering and Industrial Research, National Research Council of the Philippines, Department of Science and Technology, Philippines; d Department of Agricultural Engineering and Safety, Faculty of Engineering, Agriculture Academy, Vytautas Magnus University, Studentu str. 15A, Akademija, LT-53362, Kaunas, Distr., Lithuania

**Keywords:** Seeder, Cylindrical, Carrot, Hill center, Operating speed

## Abstract

Accurate planting of the required number of carrot seeds is one of the important parameters in developing a carrot seeding device. This study developed a metering mechanism that promotes the deposition of <3 carrot seeds on each planting hill at negligible seed damage. The metering disc and hopper were especifically designed to ensure consistency and uniformity in seed loading into the seed cell and their subsequent efficient discharge onto the planting hills. A key feature of the design is the clearance between the metering disc and the hopper, which was strategically designed to scrape excess seeds from the seed cells, allowing the surplus to freely slide back into the hopper without causing damage. The performance of the seeder was evaluated by examining key parameters, including the number of seeds dropped per hill, scattering index, hill diameter, hill spacing, and missing index across various operating speeds (60, 80, 100, 120, and 140 cm/s). The results demonstrated that the seeder achieved optimal performance at operating speeds between 80–120 cm/s, with an average of 1.70 to 1.81 seeds dropped per hill. Mean hill spacing between hills ranged from 10.98 to 13.50 cm at speeds below 120 cm/s. At a higher speed of 140 cm/s, the mean hill spacing and missing index were 23.06 cm and 53.33 %, respectively. Additionally, the scattering index decreased at a higher operating speed. The developed metering mechanism significantly enhanced the efficiency of the seeder, ensuring precise seed placement and reducing seed wastage across varying operating conditions.

## 1 Background

Carrot is one of the major crops cultivated in high-altitude areas in the Philippines [1]. It thrives well in the Cordillera Administrative Region (CAR), particularly in colder places such as in the provinces of Benguet, Mountain Province, and Ifugao, with Benguet emerging as the leading producer [2]. In 2016 the national production of carrots was 65, 987 MT [3] of which CAR shares 88.9 % of the country's production (58, 695 MT). This is followed by Central Visayas, Northern Mindanao, and Davao Region with a share of 2 % (1, 361 MT), 1.9 % (1, 227 MT), and 1.9 % (1, 241 MT), respectively [4]. In the Cordillera region, Benguet, Mountain Province, and Ifugao contribute 92.9, 6.3, and 0.9 %, respectively [4]. Within the province of Benguet, the municipalities of Buguias and Mankayan are the leading producers. In 2019, the total land area dedicated to carrot production in the country was 4, 550.90 ha to which CAR contributed around 3, 210.81 ha [3].

The mechanization level of vegetables in the Philippines, to include carrots, is low, relying primarily on human and animal labor [5]. In terms of farm mechanization, indicated by horsepower per hectare (hp/ha), several Asian countries showcase varying levels of advancement. Japan ranks first with 7.0 hp/ha, followed by South Korea at 4.11 hp/ha, China at 4.10 hp/ha, and Vietnam at 1.56 hp/ha [6]. These countries have made considerable strides in adopting mechanization in their agricultural practices. The Philippines lags with significantly lower progress of only 1.23 hp/ha. Since 1990, the country has experienced minimal improvement, with an initial mechanization level of 0.52 hp/ha [6]. The number shows a slower pace of mechanization development of the agricultural sector in the Philippines compared to its regional counterparts.

The current practice of the farmers in the Philippines presents the opportunity and need to develop a location-specific carrot seeding machine. To date the planting of carrots is accomplished manually, relying purely on manual labor and traditional tools. The tool used by the farmers is usually made of wood or light materials with protruded components equally spaced to distinguish the row spacing and spacing within the row (hill spacing). The device works by manually pushing it over the surface of the plant bed to allow the protruded components to create holes in the surface of the plant bed. The holes are then planted with seeds and then covered with soil.

The prevalent challenge of mechanical seeders is associated with seed damage and singularity of seed during the planting process. This challenge encountered in developing carrot seeders is brought by the distinct shape and characteristics of the seeds. Carrot seeds are diminutive, oval-shaped, with a gently ribbed or bumpy texture. Typically the seed measures between 1.0 and 2.0 mm in length. The lightweight nature of the seeds makes them prone to easy dispersion. Such physical properties make it difficult to plant precisely through mechanical seeders [7,8].

Seed singulation is an important parameter to achieve in planting carrots for two main reasons: one is for economic consideration and the other is to meet the planting requirement. Planting multiple carrot seeds on a hill results in seed wastage and entails economic costs. Additionally, the excess plants need to be removed to maintain a single carrot plant on a hill. This thinning operation will require additional labor costs and can damage the roots of the remaining plant. The small and irregular shape of the carrot seeds causes variability in the number of seeds filling the seed cells affecting the mechanical seeder to consistently dispense a uniform number of seeds on each planting hill [9,10].

The seeder performance is affected by several factors of the seeder design itself aside from the seed characteristics. Kumar et al. (2021) observed that the multiple index of the paddy seeder varies at different seed metering designs: 4.17 % for inclined plate, 18.45 % for cell-type metering mechanisms, and 70.42 % for fluted roller [11]. Seed damage was influenced by both the speed and design of the metering mechanism, with higher speed resulting in increased seed damage [9,11]. In the case of a cell-type seeder, considerable seed damage of 14.22 % was observed [11]. Badua et al. (2021) found that both the miss index and the multiple index were influenced by the speed of planting [12]. Yazgi et al. (2007) introduced an adjustable singulation device to reduce the multiple index by allowing excess seeds to fall back into the hopper [13]. Gaikwad et al. (2008) developed a vacuum-based seed singulation unit suitable for capsicum and tomato seeds [14]. On the other hand, various studies have explored the use of pelleting of small-sized and irregularly shaped seeds to increase manageability during planting [8,10]. These solutions have been found effective, especially the use of pneumatic seeders, in handling irregularly shaped seeds. However, the need to modify the seeds such as pelleting may be an additional cost to the farmers and could be economically impractical, especially for small-scale farms. Pneumatic seeders can plant irregular-shaped seeds [15] and can deposit the required number of seeds precisely but are often referred to in highly mechanized farms [16].

A variety of metering mechanisms have been explored in previous works. A metering disc with cells on its side was developed by Kyada et., (2014) [17]. This design offers considerable space to allow enough interaction between the seeds and the disc, especially the seed cells. Such a configuration can ensure the loading of seeds into the cell to minimize missed hills during the operation.

Khan et al. (2015) developed a seeder where the hopper was located on the upper portion of the metering disc [18]. The design has limited contact between the hopper and the disc. The loading of the seed happens when the seed cell is aligned with the bottom of the hopper. A similar design was previously developed where the hopper is configured so that it covers the top portion of the disc [19]. The minimal contact of the hopper and disc allows the hopper to be filled with seeds such that the discharge side still contains seeds. In these designs, the speed of the rotation of the metering disc has an important impact on the loading of seeds into the seed cells.

The uniformity of the number of seeds planted among the hills and seed damage due to abrasion and cutting during rotation of the metering disc is commonly encountered in mechanical seeders comprising a rotating metering disc. In particular, such a problem can be observed when the hopper is located at the top of the metering disc such that the cell will load the seed as it aligns to the hopper and then eventually goes to the discharge point upon rotation. This configuration has minimal contact between the seed cell and the hopper which encourages multi and under-loading of seeds. Such a design offers low interaction time between the seed cell and the quantity of seeds contained in the hopper. Due to this, the variability in the number of seeds loaded into the seed cells during rotation is high. On the other hand, the slicing of seeds can easily occur since there is a tight clearance between the metering disc and the hopper.

This study was performed to address the high variability in the number of seeds planted on each hill and to come up with an alternative design to minimize seed damage during the rotation of the metering disc. The operational efficiency of the seeder was assessed in terms of seed count per hill, center-to-center hill spacing, scattering index, and missing index following established methodologies as outlined in previous studies [15,20,21] under varying forward operating speeds in a laboratory condition.

## 2 Materials and methods

### 2.1 Carrot seed

Carrot seed (Tokita Kuroda) purchased from a local farm supply was used in the evaluation. The seed properties were characterized as outlined in our previous research, detailed in Table 1 [22]. These physical attributes include length (l), width (w), thickness (t), sphericity (Φ) and geometric mean diameter ($\phi_{GM}$). The sphericity [13,23] and geometric mean diameter [15,24] were determined using Eq. (1) and Eq. (2), respectively.

(1) $$\Phi =\frac{{\left(lwt\right)}^{1/3}}{l}x100\%$$

(2) $$\phi_{GM}={\left(lwt\right)}^{1/3}$$

**Table 1:** The physical properties of Tokita Kuroda carrot seeds used in the evaluation of the seeder.

Physical properties	Mean	Standard Deviation	Coefficient of Variation, %
Length, mm	3.85	0.04	1.10
Width, mm	1.72	0.02	1.09
Thickness, mm	0.80	0.03	4.34
Φ, %	45.32	0.41	0.90
$\phi_{GM}$, mm	4.63	4.63	2.09

### 2.2 Metering assembly

**Fig. 1:**
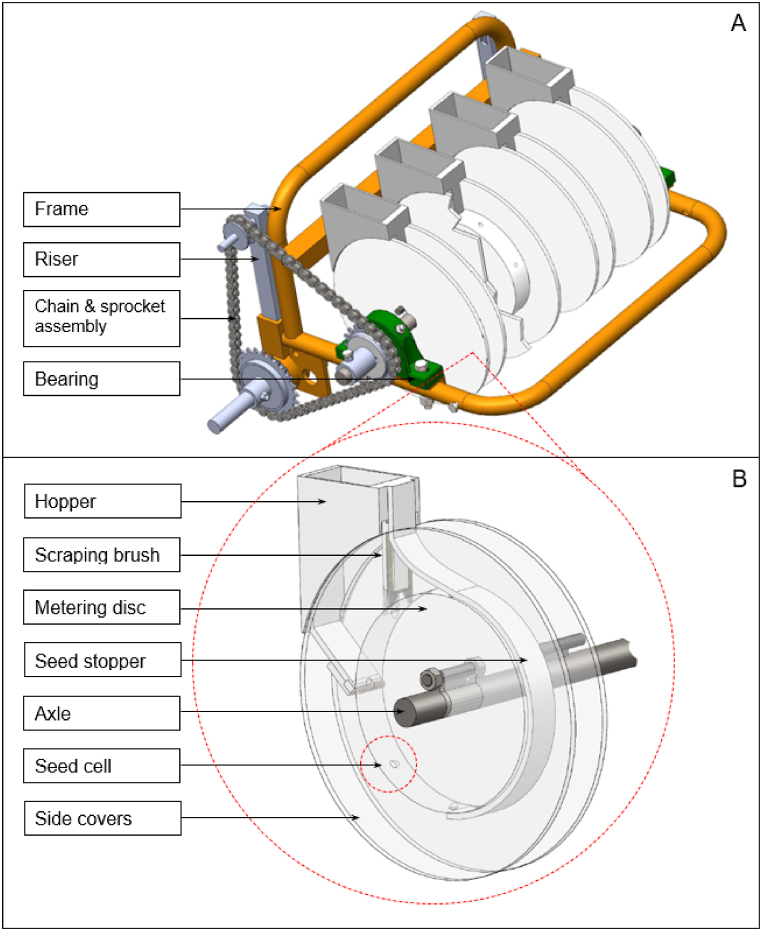
The metering assembly (A) and details of the hopper and metering disc (B) of the carrot seeder.

The main components of the metering assembly are hopper, chain, and sprocket, metering discs synchronized and held through an axle, and the frame shown in Fig. 1A. The metering disc, 2.54 cm thick and 250 cm in diameter, was grooved (seed cell) with 8 holes around the circumference (Fig. 1B). These grooves are the seed cells and each cell was designed to accommodate at least 2 to 3 seeds and is inclined to facilitate seed loading and discharge. The metering disc is sandwiched by two circular discs (side cover) that serve as casing and form part of the hopper. The side cover discs are made of transparent material to allow easy monitoring of seed movement. These sandwiching discs have thickness and diameter of 1.0 cm and 300 cm, respectively and they were tightly fastened to the metering disc through bolts and nuts. The part of the hopper at its exit point is provided with a scraping brush to prevent the seeds from shearing. A seed stopper is provided at the discharge portion of the metering disc. The seed stopper covers the half portion of the metering disc to hold the seeds in the cell during rotation. With this, the seeds will only fall once the seed cell reaches the bottom portion during rotation. This is to ensure uniform distribution of seeds along the plant bed.

**Fig. 2:**
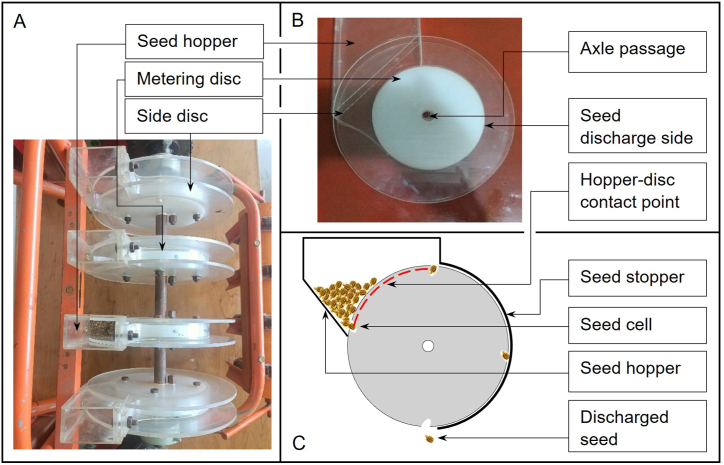
The fabricated metering assembly of the carrot seeder. A) Actual photo of the metering assembly; B) Side view of the metering disc showing the side discs and hopper; C) The schematic diagram for the seed movement during loading and unloading from the seed cell.

Fig. 2A shows the fabricated metering assembly of the carrot seeder. The half part of the circumference of the metering disc is covered with a flexible material serving as a seed stopper to maintain the seed in the seed cell as the disc rotates until it reaches the discharge point where the seed will freely fall into the ground. The bottom of the hopper is V-C shaped where one part is inclined at 45° and the other is in C-shape which is in contact with the metering disc, shown in Fig. 2B and C. This design permits surplus seeds within the seed cell to naturally return to the lower section of the hopper. The angled bottom serves the purpose of easing the process of depositing seeds into the seed cells.

### 2.3 The seed cell

**Fig. 3:**
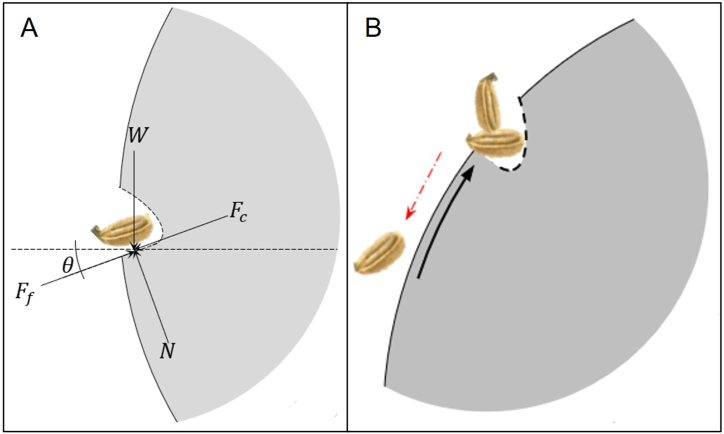
The seed cell loaded with carrot seed illustrates the free body diagram of the seed (A); and the scenario where excess seed naturally falls back into the hopper (B).

The geometric design of the cell is shown in Fig. 3. The cell's bottom section is inclined at 20°, corresponding to the angle of repose of the carrot seed, calculated using Eq. (3) following the previous approach described in Ref. [22]. This inclination facilitates regulated seed loading and allows any excess seeds to slide back into the hooper if multiple seeds are loaded. Initially, seeds are loaded into the cell when it is positioned at the bottom of the hopper, as shown in Fig. 3A. In some instances, the cell may load multiple seeds, especially small seeds. Still, the design allows for regulation enabling excess seeds to return to the hopper when the disc rotates, as depicted in Fig. 3B. The size of the metering disc usually affects the scenario where the excess seed will fall back into the hopper.

(3) $$\theta =\sin^{-1}\left(\frac{F_{f}-F_{c}}{\mu \;mg}\right)$$

Where F_c_ represents the centrifugal force generated by the cylinder's rotation (in Newton, N), F_f_ denotes the force of friction opposing the movement of the seed (N), θ is the angle of inclination of the seed cell, μ corresponds to the unitless static friction coefficient (SFC) that characterizes the interaction between the seed and the cylinder, m is the mass of the seed, and g is the acceleration attributed to gravity (m/s^2^). The frictional force is the main factor that prevents the seeds from sliding from the seed cell into the ground which can be modified by adjusting the value of θ. The SFC was determined with the use of a plane with adjustable inclination as described in Refs. [22,25,26]. The value for u was calculated using Eq. (4) [27].

(4) u=tanθ

**Fig. 4:**
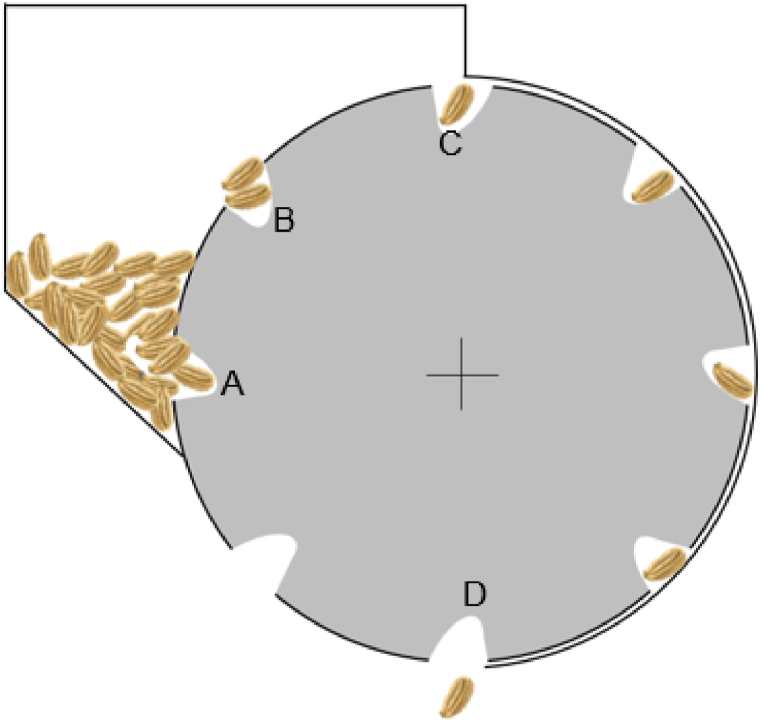
The metering mechanism of the seeder, depicting the seed loading at cell A, and seed dispensing at cell D.

The angle θ is a crucial parameter in preventing the seeds from dropping off the cell [19]. For effective seed loading, the slant height (sh) should be slightly shorter than the seed length, and sh is a function of θ. The effectiveness of seed loading and the ability of multi-layered seeds to return to the hopper can be significantly influenced by the diameter of the metering disc, as illustrated in Fig. 4. When the metering disc has a small diameter, the distance between points A and C is reduced. This decreases the available distance and time for excess seeds to be expelled into the hopper from a multi-seed load at point B. Consequently, the only opportunity for excess seeds to be discarded occurs between points B and C.

**Fig. 5:**
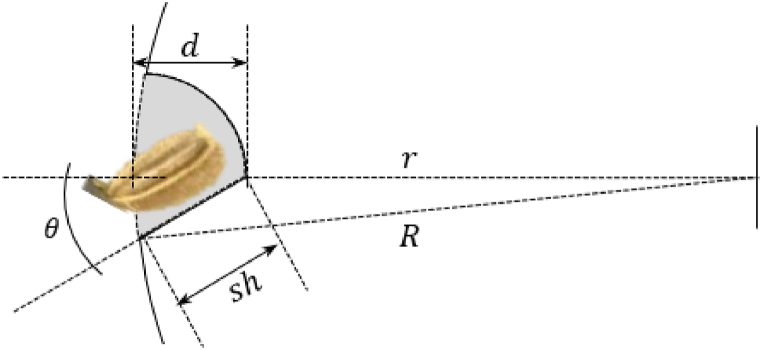
The variables comprising the geometric figure of the seed cell.

Fig. 5 illustrates the geometric details of the seed cell, including the cell depth, (d), slant height inclined at an angle θ, and outside radius (R) of the metering disc. Additionally, the figure shows the inside radius (r) of the geometry of the metering disc in relation to the depth of the seed cell.

Utilizing the law of cosine, Eq. (5) describes the relationship between the parameters of the seed cell shown in Fig. 5. Specifically, the minor radius, denoted as r (mm) is represented as the difference of the outer radius R from the cell's depth d, expressed as (R−d) both in mm. The sh is designed to be less than the average length of the seed, within 50 %–80 % (0.5l<sh<0.8l) of the seed length [22], which is 3.85 mm, as detailed in Table 1 and was calculated according to Ref. [28]. The depth of the cell was 80 % of the seed length (d=0.8l). The number of cells was calculated using Eq. (6) where n represents the number of seeds, R denotes the radius of the metering, and HS corresponds to the designed hill spacing for carrots, specified as 10.0 cm.

(5) $$R^{2}={sh}^{2}+r^{2}-2r\left(sh\right)\cos\left(180-\theta \right)$$

(6) $$n=\frac{\pi 2R}{HS}$$

### 2.4 Evaluation

The assessment of the seeder encompassed trials at five distinct operational velocities, as outlined in (Table 2). The testbed utilized for these evaluations measured 1000.0 cm in length, and 75.0 cm in width, and stood at a height of 14.0 cm. To avoid seed displacement, the test bed surface was coated with grease [10,29]. The evaluation followed the protocols set forth by the Agricultural Machinery Testing and Evaluation Center (AMTEC) [30].

**Table 2:** Experimental design for evaluating the carrot seeder at various operating speeds (OS).

Treatments	Levels
Operating speeds (nominal), cm/s	OS_1_ = 60
	OS_2_ = 80
	OS_3_ = 100
	OS_4_ = 120
	OS_5_ = 140
Replication	3.0
Fall height, cm	14.0
Bed length, cm	1000.0
Bed width, cm	75.0

**Fig. 6:**
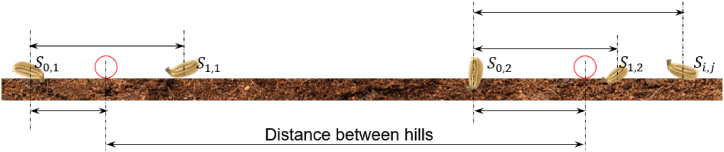
Schematic diagram of seed placement on the plant bed, highlighting the location of the seeds relative to the first seed dropped on each hill.

The hill-dropping uniformity was assessed following the previously described methodology [19,[31], [32], [33]]. The relative distances between seeds within a hill were measured to determine the hill center and the scattering index [19,33]. The positions of dispersed seeds within a hill were determined reckoned to the first seed that dropped, denoted as ($S_{i,j}=S_{0,1}=S_{0,2}$), as illustrated in Fig. 6. Each seed's location is labeled as $S_{i,j}$, where the subscripts i and j represent the $i^{th}$ seed within the $j^{th}$ hill, as defined in Ref. [22].

**Fig. 7:**
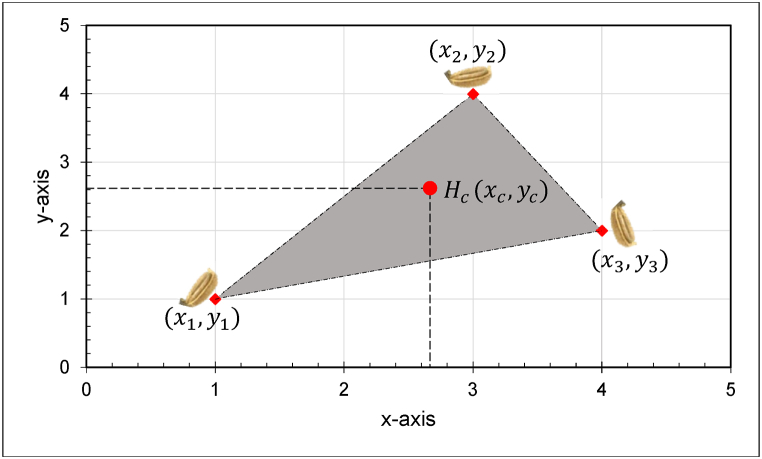
The schematic diagram for the determination of center of multiple seeds dropped on a hill.

Furthermore, the center of the seeds on a hill was calculated with the aid of the illustration in Fig. 7. The center of the hill was determined by taking the average distance of the seeds to the x-axis and y-axis. Thus the coordinate of the center of the seeds is $\left(x_{c},y_{c}\right)=\left(2.66,2.66\right)$. Considering that the seeds are in one line, then the y component of the coordinate could be removed for simplicity. Hence the hill center is calculated with the use of Eq. (7).

(7) $$H_{c}=\frac{S_{1,x}+S_{2,x}+S_{n,x}}{n}$$

Where, $H_{c}$ is the hill center (cm); $S_{n,x}$ is the distance of the seed $n^{th}$ to the origin along the horizontal axis; and n is the number of seeds. The origin can be identified arbitrarily. It could be the first seed on the hill. The circle that encloses the seeds within a hill defines the hill's diameter. The distance between consecutive $H_{c}$ points represent the hill spacing.

### 2.5 Scattering index

**Fig. 8:**
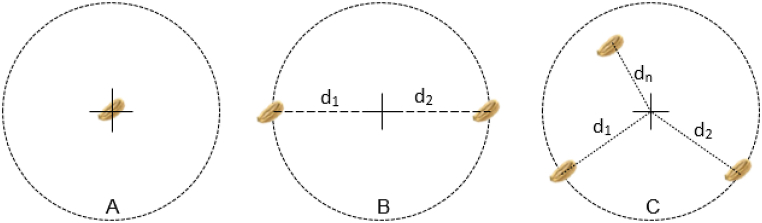
The seeds dropped onto a hill: A), a single seed on a hill; B), a double seed on a hill; and C), several seeds on a hill.

In the absence of an established equation, the following is proposed for calculating the scattering index (SI) (Eq. (8)). Here, SI, is the scattering index; di, is the distance of the seed to the hill center; and n, is the number of seeds in the $j^{th}$ hill. This calculation is based on the principle of area moment. As illustrated in Fig. 8A, when a single seed is dropped in a hill, by default it's position is the center of the hill. In the case of two seeds, the center is the midpoint of the line connecting the seeds (Fig. 8B). The scattering index is determined by calculating the area moment, which is the product of the area occupied by each seed and its distance from the hill center. The sum of these area moments yields the scattering index. Although the seed occupies only a small area, it is not negligible; for simplicity, the seed area is assigned a value of 1.0. A higher SI value indicates greater dispersion of seeds within the hill compared to lower SI values.

(8) $$SI=\sum_{i=0}^{i=n}\left(S_{ij}*\;d_{i}\right)$$

## 3 Results and discussion

### 3.1 The newly developed carrot seeder

**Fig. 9:**
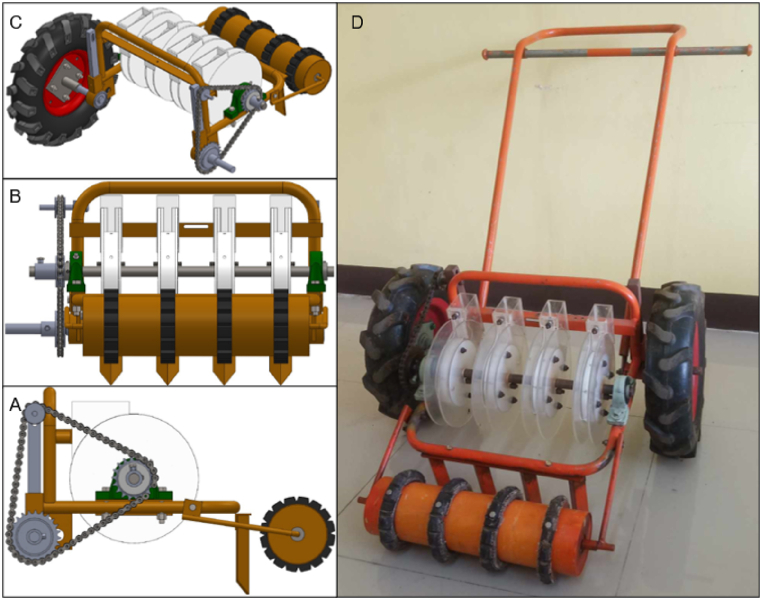
The developed carrot seeder: A) transmission mechanism; B) furrow opener with a front wheel to control the depth of furrow; C) metering assembly; and D) fabricated carrot seeder.

Fig. 9D shows the designed and fabricated carrot seeder comprising a ground wheel; metering assembly; chain and sprocket mechanism; frame; and handle. The metering system has four metering discs, firmly affixed to an axle running through their centers. These discs are held in place by ball bearings at both ends of the axle. A chain and sprocket mechanism is integrated into the device to transmit rotational energy from the ground wheel to the metering discs (Fig. 9A). Additional features of the seeder include front wheel and furrow openers (Fig. 9B). The furrow opener is provided to create corrugation on the plant bed for the seed placement. The attached roller wheel at the front of the seeder controls the depth of the corrugation. Likewise, it balances the seeder during operation. The frame that connects to the ground wheel is adjustable (Fig. 9C). This is to make the clearance of the seeder to the plant bed adjustable.

The performance of the seeder assessed across five operating speeds is summarized (Table 3). The average number of seeds dropped per hill and the missing index was significantly affected by the operating speed (p < 0.05). However, the hill diameter, hill spacing, and scattering index were not affected by the operating speed.

**Table 3:** Summary of the performance of the seeder.

Performance parameters	p values
Average number of seeds in each hill	0.00049∗
Hill Diameter, cm	0.172 ^ns^
Hill spacing, cm	0.566^ns^
Scattering index	0.462^ns^
Missing index, %	0.007∗

^Ns^,∗, nonsignificant or significant at the p-value ≤0.05, respectively.

### 3.2 Number of seeds

The average number of seeds planted on each hill ranged from 0.70 to 1.81 (Table 4). This conforms with the findings reported in the AMTEC Test Report No. 2020-0448 [34] and AMTEC Test Report No. 2021-0025 [30]. The analysis of variance (ANOVA) shows statistical significance (p = 0.00049) in the variation of the average number of seeds per hill, attributable to differences in operating speeds. The least significant test (LSD) test showed that at a speed of 120 cm/s and below, the seeder was able to drop statistically the same number of seeds in each hill. Conversely, when the seeder was operated at 140 cm/s, the average number of seeds significantly decreased to 0.70. Further analysis showed that the standard deviation (SD) and coefficient of variation (CV) were highest at 140 cm/s with values of 2.51 and 0.42, respectively. The high-speed induced collision and bounce from the seed cylinder [35] caused surface instability in seed loading [36] which potentially reduced the time available for seeds to load into the cell. This, in turn, has led to a decrease in the number of seeds planted on each hill.

**Table 4:** The influence of operating speed on the number of seeds placed in each hill, the average hill diameter, and the spacing between hills.

Operating speed (cm/s)	Average seeds per hill	Hill diameter (cm)	Spacing between hills (cm)
60	1.70^a^	1.67^a^	12.18^a^
80	1.81^a^	1.43^a^	13.50^a^
100	1.70^a^	1.30^a^	13.35^a^
120	1.74^a^	1.75^a^	10.98^a^
140	0.70^b^	0.86^b^	23.06^b^

### 3.3 Hill diameter

In the case of the hill diameter (Table 4), when the seeder was operated at 60 to 120 cm/s, the difference in the hill diameter is statistically insignificant with values ranging from 1.30 to 1.75 cm. However, at a higher operating speed of 140 cm/s, the hill diameter significantly decreased (0.86 cm). The hill diameter indicates the dispersion of the seeds in a hill but this is more appropriately discussed in the scattering distance index section. The higher hill diameter indicates that seeds in a hill are relatively far from each other. Conversely, a lower hill diameter would mean that the seeds are close to each other. In the case of a single seed dropped on a hill, the hill diameter is zero.

### 3.4 Spacing between hills

Table 4 shows the spacing between the hill. When the seeder was operated at 60 to 120 cm/s, the differences in hill spacing were statistically insignificant and remained within the designed spacing of 10.0 cm. However, at 140 cm/s, the hill spacing increased significantly (23.06 cm). Plant spacing is a major parameter of a seeder [37]. One factor of large spacing between hills could be that the metering disc failed to load seeds or it could be that it loaded seeds but it failed to drop. The scenario where seeds fail to drop could be associated to the sticky property of the carrot seeds caused by their hairy structure. In addition, at a relatively fast rotation of the metering disc, the seeds in the cell have not enough chance to fall into the ground. When this happens, the cell will load more seed as it returns to the hopper. Because of this, the seeds in the cells are forcedly fitted which impedes free fall motion. The seeds in the cell can either be released into the hopper or stay in the cell. The cells can load additional seeds causing multiple seeds. In some instances, due to the existing seeds in the seed cell, there is a possibility of the seed being forcedly loaded into the seed causing a crowd in the cell. At this point, it is more difficult for the seeds to drop into the ground and there is a need to manually remove such seeds out of the cell. On the other hand, the increased speed caused a delay and variability in the discharging of seeds affecting the spacing of the dropped seeds.

### 3.5 Scattering index

The scattering index, which measures the dispersion of the seeds in a hill, is shown in Table 5. For carrots, planting seeds too close together is disadvantageous, as it necessitates a thinning operation to remove excess plants, typically performed 2–3 weeks after planting. This process risks damaging the roots of the plants that are meant to be retained. In precision seeding, minimizing seed scatter is beneficial [31]. An SI lower than 30 % is acceptable in precision hill dropping in crops like rice [19]. The SI values at OS 80 to 120 cm/s are statistically the same. However, the SI significantly decreased to 3.47 and 5.93 % at 140 and 60 cm/s, respectively. This suggests that operating the seeder at a high OS like 140 cm/s will result in low SI, primarily because the seeder drops fewer seeds per hill at this speed [22], compared to lower OS where more seeds are planted. On average, only 0.70 seeds were dropped per hill (Table 4) with the lowest SI (3.47) observed at 140 cm/s. However, this low SI does not necessarily indicate optimal performance, as the reduced number of seeds dropped per hill—sometimes even just a single seed—contributes to the low SI, potentially compromising the effectiveness of planting at higher speeds.

**Table 5:** The performance of the seeder in terms of the scattering distance index of the seeds and missed hills as affected by the operating speeds.

Operating speed (cm/s)	Scattering index	Missed hills %
60	5.93^b^	23.33^a^
80	12.25^a^	16.67^a^
100	11.65^a^	26.67^a^
120	9.16^a^	10.00^a^
140	3.47^b^	53.33^b^

### 3.6 Missing index

The impact of operating speed on missed hills is shown in Table 5. The results indicate that operating speeds between 60 and 120 cm/s did not significantly affect the number of missed hills. However, at 140 cm/s a significant increase in the missed hills was observed. A hill is considered missed when the spacing between two consecutive hills exceeds 1.5 times the designed spacing [11,13]. The peripheral speed affects the missing index of a seeder [13]. A similar observation was reported from a vacuum seeder for maize and soybean where a miss index of 28.2 % was high at a higher forward speed (2.0 m/s) [38]. Additionally, a direct paddy seeder had the highest missing index of 52.73 % at 3.5 kph compared to 31.91 % at 2.5 kph [11]. The high missing index is associated with the failure of the seeder to drop seeds [21], especially at high operating speeds. Gautam et al. (2019) attested that the miss index tends to rise with an increase in operating forward speed [10]. The scenario in which a high OS such as 140 cm/s gave a high missing index could be due to the seeds being put into random and fast motion brought by the high rotation of the metering disc. The fast rotation causes the seeds to avalanche within the hopper [22,39], disrupting their flow and reducing the time available for seeds to load into the cells properly. As a result, the spacing between hills at 140 cm/s increased to 23.06 cm—more than twice the theoretical spacing of 10 cm—indicating that some hills were indeed missed at this speed.

## 4 Conclusions

In this study, a portable carrot seeder was developed. The seeder was designed with a metering mechanism where the hopper was configured to cover a quarter of the metering disc which provides enough interaction time of the seeds and seed cells during rotation. This design enables the metering disc to accurately load carrot seeds while allowing excess seeds to return to the hopper. The performance of the seeder was assessed in terms of the number of seeds planted per hill, hill diameter, spacing between hills, scattering index, and the missing index. These evaluations were carried out at different operating speeds: 60, 80, 100, 120, and 140 cm-s^−1^. The missing index was minimum at 120 cm/s at 10 % while it was highest at 140 cm/s. The number of seeds planted in each hill was optimum at 120 cm/s and below while it decreased at 140 cm/s due to missing hills. The findings suggest that the optimal operational range for the seeder falls within speeds of 80–120 cm-s^−1^. This study presents a promising opportunity to enhance the existing problems on multi-seed index, and seed damage of mechanical seeders.

## CRediT authorship contribution statement

**Marvin T. Valentin:** Writing – original draft, Investigation, Software, Data analysis, Funding acquisition, Conceptualization, Supervision. **Keithler M. Pagnas:** Investigation, Methodology, Data acquisition, Data curation, Software, Validation. **Roger Lee M. Suclad:** Investigation, Methodology, Data acquisition, Data curation, Software, Validation. **Algirdas Jasinskas:** Investigation, Validation, Writing – review and editing, Funding acquisition. **Rolandas Domeika:** Investigation, Writing – review and editing. **Egidijus Šarauskis:** Investigation, Writing – review and editing.

## Availability of data and materials

The data used in this study is available to the corresponding author upon request.

## Declaration of competing interest

The authors declare the following financial interests/personal relationships which may be considered as potential competing interests: Marvin T. Valentin reports financial support was provided by the 10.13039/501100010892Technology Application and Promotion Institute-Department of Science and Technology (DOST-TAPI), Philippines.
